# Patients’ willingness to pay for cognitive pharmacist services in community pharmacies

**DOI:** 10.3325/cmj.2017.58.364

**Published:** 2017-10

**Authors:** Dragana Lakić, Ivana Stević, Marina Odalović, Sandra Vezmar-Kovačević, Ivana Tadić

**Affiliations:** 1 Department for Social Pharmacy and Pharmacy Legislation, University of Belgrade, Faculty of Pharmacy, Belgrade, Serbia; 2 Department for Pharmacokinetics and Clinical Pharmacy, University of Belgrade, Faculty of Pharmacy, Belgrade, Serbia

## Abstract

**Aim:**

To determine the general population willingness to pay for cognitive pharmacist service in community pharmacy, describe the behavior of participants regarding health care issues, and evaluate correlation between participants’ sociodemographic characteristics or attitudes and their willingness to pay.

**Methods:**

A questionnaire-based survey was conducted among general population visiting community pharmacies. The participants were asked about receiving cognitive pharmacist services to identify and resolve potential medication therapy problems after the initiation of a new medicine to optimize health outcomes of the patients. A univariate and multivariate analysis were used to analyze associations between different variables and willingness to pay for pharmacy service.

**Results:**

Of 444 respondents, 167 (38%) reported that they were willing to pay for a medication management service provided in the community pharmacy. Univariate analysis showed significant association between the willingness to pay for pharmacist-provided service and respondents’ socio-demographic factors, health-related characteristics, and behavior, dilemmas, or need for certain pharmacist-provided service. The logistic regression model was statistically significant (χ2 = 4.599, *P* < 0.001).

**Conclusions:**

The respondents expressed their willingness to pay for cognitive pharmacist services, which has not been fully recognized within the health care system. In future, pharmacists should focus on practical implementation of the service and models of funding.

Pharmacists play a unique role in contributing to health outcomes of patients and improvement of patient’s quality of life due to accessibility of a pharmacy and pharmacist, traditional activity of supply and dispensing medicines, and pharmacy services related to the pharmacists’ use of specialized knowledge and abilities to help patients achieve effective and safe pharmacotherapy (1). Patient-oriented pharmacy services have been associated with the improvement of clinical and economic outcomes, quality of life, patient safety, and reduction in morbidity and mortality (1-3).

Today, almost every health service or program needs to be evaluated in economic terms, justifying that the allocated money is worthwhile. Cost-benefit analysis is a type of economic evaluation where both costs and outcomes of a certain health care program are expressed in monetary terms. One of the usually applied techniques to perform monetary valuation in cost-benefit analysis is willingness to pay, which can be assessed using the revealed or stated preferences approach, also known as contingent valuation. Contingent valuation uses survey methods to present participants with hypothetical scenarios about the health care program (4).

The same principles apply for the cognitive pharmacy services. Evaluation of the willingness to pay for different pharmacy services in developed countries showed that the general population was willing to pay for the cognitive service provided in community pharmacies, where the amount was highly dependent on the service provided, but in certain cases reached more than US$30 (5-8). In less developed countries, willingness to pay for services in pharmacy was rarely evaluated, mainly because of the focus on traditional services, such as dispensing (5). Cognitive pharmacy service, like medication therapy management, differs from counselling during the dispensing of medicines because it includes comprehensive and overall review of patient’s medicines, dietary regime, and issues related to the medications usage (1).

The primary aim of this study was to determine general population’s willingness to pay for a cognitive pharmacist service in community pharmacies in Serbia. The secondary aims were to evaluate behavior of participants regarding the health care issues and explore the predictors of willingness to pay and behavior.

## PARTICIPANTS AND METHODS

A cross-sectional survey was conducted in community pharmacies in Serbia from April 25, 2016 to May 25, 2016.

### Participants

Participants’ recruitment occurred at the counter when a customer approached the pharmacist. The pharmacist invited customers to fill out a questionnaire at the counter. All customers entering the pharmacy were invited irrespective of their reason for visiting the pharmacy, possession of prescription, or specific service needed.

Participating pharmacies were selected to reflect the number and distribution of the pharmacies in Serbia. The sample of pharmacies was selected on the basis of a previous study investigating community pharmacy network in Serbia (6) and included 70 community pharmacies, either publicly or privately owned. Each pharmacy received 10 printed questionnaires to be distributed to the pharmacy customers.

### Method

Pharmacy customers were asked to fill out the anonymous questionnaire inquiring about their demographic and health-related characteristics, behavior, the need for pharmacy service, and willingness to pay for cognitive pharmacist service. The written explanation of the service included brief description of the possible counselling as follows: oral and written counselling and activities performed by the pharmacist to identify and resolve potential medication therapy problems after the initiation of a new medicine to optimize health outcomes of the patients, such as interactions with current medications or diet, possible adverse effects, and what to expect from the treatment.

The participants’ willingness to pay was measured through close-ended binary choice question (yes/no). If the respondents answered “yes” to the question about the willingness to pay, they were also asked to choose one of the defined values for the service. There were five defined values as follows: less than US$0.5, US$0.5-1, US$1-2.3, US$2.3-4.6, more than US$4.6. The values are linked with the Serbian health insurance system, where usual co-payment for the dispensed package of medicine is US$0.5. Serbia has social health insurance with compulsory funding through employers and employees contribution. The average gross salary in 2016 was US$567.9 (7), from which 10.3% deduction were allocated to health insurance. Participation in the survey implied the consent by the respondents.

### Questionnaire

Dependent variable was willingness to pay for cognitive pharmacist service coded as “yes” or “no”, where ”no“ was considered as a reference category.

The independent variables considered as potential predictors of willingness to pay for cognitive pharmacist provided service included socio-demographic characteristics, health-related characteristics and behavior, and dilemmas and need to certain pharmacist service. Socio-demographic characteristics included age (15-24, 25–34, 35-44, 45-54, 55-64, and ≥65 years), gender (male/female), education (primary, secondary, or graduate level), employment status (unemployed, employed, or retired), health care professional (no/yes), and internet use (no/yes). Health-related characteristic and behavior of respondents included the frequency of pharmacy visiting (once a week, once in two weeks, once a month, or once in several months), number of pharmacies for medicine supply (1, 2-3, or >3), source of information on medicines (physician, pharmacist, friends and family, internet, or several of the above), presence of chronic disease (no/yes), and medication therapy for chronic disease (no/yes). Dilemmas and need for the certain pharmacist service included need for written information from pharmacist (no/yes), dilemmas regarding the discontinuation of medication therapy due to adverse effect (no/yes), and dilemmas regarding the adequate medication administration and use (no/yes).

### Statistical analysis

Descriptive statistical analysis including frequencies was used to present the study sample characteristics. A univariate analysis was used to analyze univariate associations between independent (potential explanatory) variables with willingness to pay for pharmacy service. Crude odds ratio (cOR) with 95% confidence interval (CI) was used to explain the association of explanatory variable and willingness to pay. Variables found to be associated with medication use in the univariate analysis were included in the multivariate logistic regression model. The impact of variables on willingness to pay was estimated as adjusted odds ratio (aOR) with 95% CI. Hosmer-Lemeshow test of the goodness-of-fit was used to determine the goodness-of-fit of the model to the data.

Missing values were excluded from the analysis. The level of statistical significance was set at *P* < 0.05. Data analysis was performed using Statistical Package for Social Science (SPSS) software (SPSS 18.0 for Windows, SPSS Inc., Chicago, IL, USA).

## RESULTS

The total number of respondents who completed the questionnaire was 444 .(response rate 63.4%). The mean age of participants was 45.1 ± 16.1 years, with 37.1% of survey respondents being male. There was an almost equal number of respondents with secondary or graduate level education, and 18.5% of respondents had medical background (Table 1).

**Table 1:** Socio-demographic characteristics of survey respondents

		No. (%) of respondents*	
Socio-demographic characteristic	total (N = 444)	willing to pay (n = 167)	not willing to pay (n = 277)
Age group (years)			
15-24	24 (5.4)	6 (1.4)	18 (4.1)
25-34	121 (27.4)	57 (12.9)	64 (14.5)
35-44	84 (19.0)	30 (6.8)	54 (12.2)
45-54	70 (15.9)	19 (4.3)	51 (11.6)
55-64	79 (19.7)	35 (7.9)	44 (9.8)
≥65	63 (14.3)	20 (4.5)	43 (9.8)
Gender			
male	160 (37.1)	52 (12.1)	108 (25.1)
female	271 (62.9)	111 (25.8)	160 (37.1)
Education level			
primary	23 (5.2)	5 (1.1)	18 (4.1)
secondary	210 (47.5)	66 (14.9)	144 (32.6)
graduate	209 (47.3)	95 (21.5)	114 (25.8)
Employment status			
unemployed	82 (18.5)	27 (6.1)	55 (12.4)
employed	267 (60.3)	104 (23.5)	163 (36.8)
retiree	94 (21.2)	35 (7.9)	59 (3.3)
Healthcare professional			
no	357 (81.5)	121 (27.6)	236 (53.9)
yes	81 (18.5)	42 (9.6)	39 (8.9)
Internet user			
no	100 (23.1)	37 (8.5)	63 (14.5)
yes	333 (76.9)	125 (28.9)	208 (48.0

*Numbers do not add up due to missing values: age (3); gender (13); education (2); employment status (1); health care professional (6) and internet user (11).

A chronic disease, such as hypertension, diabetes, pulmonary disease, or osteoporosis, was present in 39.3% of respondents. About a third (32.7%) of the respondents reported visiting a pharmacy once a month. The primary reasons for visiting a certain pharmacy were location of the pharmacy (27.5%), price of the products (27.1%), pharmacist service (23.1%) or pharmacy staff (21.7%). Physician and pharmacist were the respondents’ primary and secondary sources of information on medicines or medical conditions (38.1% and 25.7%, respectively). Almost one quarter (23.4%) of the respondents did not contact any medical personnel for health-related information (Table 2).

**Table 2:** Health-related characteristics and behavior of respondents

		No. (%) of respondents*	
Health-related characteristic	total (N = 444)	willing to pay (n = 167)	not willing to pay (n = 277)
Frequency of pharmacy visiting			
once a week	111 (25.0)	54 (12.2)	57 (12.8)
once in two weeks	118 (26.6)	41 (9.2)	77 (17.3)
once a month	145 (32.7)	52 (11.7)	93 (20.9)
once in several months	70 (15.8)	20 (4.5)	50 (11.3)
Number of pharmacies visited on regular basis			
1	163 (37.2)	60 (13.7)	103 (23.5)
2-3	217 (49.5)	83 (18.9)	134 (30.6)
>3	58 (13.2)	20 (12.3)	38 (8.7)
Source of information on medicines and medical conditions			
physician	169 (38.1)	53 (11.9)	116 (26.1)
pharmacist	114 (25.7)	56 (12.6)	58 (13.1)
friends and/or family	49 (11.0)	16 (3.6)	33 (7.4)
internet	55 (12.4)	19 (4.3)	36 (8.1)
several of above	57 (12.8)	23 (5.2)	34 (7.7)
Chronic disease			
no	269 (60.7)	109 (24.6)	160 (36.1)
yes	174 (39.3)	57 (12.9)	117 (26.4)
Medication therapy for chronic diseases			
no	275 (62.4)	108 (24.5)	167 (37.9)
yes	166 (37.6)	57 (12.9)	109 (24.7)

*Numbers do not add up due to missing values; number of pharmacies visited (6); chronic disease (1); medication therapy for chronic disease (3).

A total of 30.9% of respondents expressed the need for detailed written information by pharmacist regarding the adequate medication usage and application (Table 3). A higher percentage of participants expressed dilemmas regarding the discontinuation of the therapy due to the adverse effect of medicine or adequate medications usage (48.0% and 46.3%, respectively).

**Table 3:** Respondents’ dilemmas and need for the certain pharmacist service

		No. (%) of respondents*	
Dilemmas and needs	total (N = 444)	willing to pay (n = 167)	not willing to pay (n = 277)
Need for written information from pharmacist			
no	307 (69.1)	96 (21.6)	211 (47.5)
yes	137 (30.9)	71 (16.0)	66 (14.9)
Dilemmas regarding the discontinuation of medication therapy due to adverse effects			
no	231 (52.0)	71 (16.0)	160 (36.0)
yes	213 (48.0)	96 (21.6)	117 (26.4)
Dilemmas regarding the adequate medication administration and use			
no	237 (53.7)	69 (15.6)	168 (38.1)
yes	204 (46.3)	96 (21.8)	108 (24.5)

Note: Numbers do not add up due to missing values; dilemmas regarding the adequate medication administration and use (3).

The willingness to pay for a medication management service provided in the community pharmacy was expressed by 167 (38%) respondents. Almost equal percentage of respondents indicated a value for service of up to US$1 (31.1% of respondents willing to pay for service), between US$1 and US$2.3 (29.3%) or between US$2.3 and US$4.6 (28.1%).

A multivariate logistic regression was performed to ascertain the effects of variables significantly associated in univariate analysis on the likelihood that participants were willing to pay for cognitive pharmacist provided service. The logistic regression model (χ^2^ = 4.599, *P* < 0.001) explained 17.3% (Nagelkerke *R^2^*) of the variance and correctly classified 70.0% of cases. Hosmer-Lemeshow test *P*-value was >0.05.

Respondents with undergraduate level of education were 5.95 times more likely to pay for cognitive pharmacist provided service than participants with primary level education. Those who had medical background were 1.84 times more likely to pay than those without medical background. Additionally, using a pharmacist as the main source of medical information was associated with an increased likelihood of willingness to pay for cognitive pharmacist provided service, as well as the need for written information from pharmacist and the need for additional information about drug administration and use. The frequency of pharmacy visits and need for medication therapy discontinuation due to adverse effect were revealed as confounders in the multivariate analysis (Table 4).

**Table 4:** Relationship between respondents’ characteristics and their willingness to pay for pharmacy service*

Variable	aOR (95% CI)
Education	
primary school	reference
secondary school	3.33 (0.91-12.18)
undergraduate studies	5.95 (1.61-22.02)^†^
Healthcare professional	
no	reference
yes	1.84 (1.07-3.17)^†^
Frequency of pharmacy visiting	
once a week	1.84 (0.89-3.78)
once a two weeks	1.05 (0.52-2.13)
once a month	1.15 (0.59-2.24)
once a several months	reference
Source of data about medicines	
physician	reference
pharmacist	1.89 (1.10-3.24)^†^
friends and/or family	1.39 (0.66-2.93)
internet	1.01 (0.50-2.03)
several of above	1.12 (0.55-2.22)
Need for written information from pharmacist	
no	reference
yes	1.86 (1.15-3.01)^†^
Need for medication therapy discontinuation due to adverse effect	
no	reference
yes	1.39 (0.88-2.20)
Need for additional information about drug administration and use	
no	reference
yes	1.58 (1.00-2.52)^†^

*All variables showing univariate association with the outcome entered the model and were retained regardless of their multivariate (independent) association with the outcome. Hosmer-Lemeshow test *P* > 0.05. aOR - adjusted odds ratio, CI - confidence interval.

†*P* < 0.05

## DISCUSSION

We found that more than a third of the respondents were willing to pay for a pharmacy service aimed at optimizing therapeutic outcomes for a specific patient after the initiation of the new medicine.

Pharmacist counselling was shown to have positive effect on patients’ drug and therapy knowledge, therapy adherence, quality of life, reduction of medication-related problems, and demonstrate positive economic outcomes (8,9). Previous studies showed that between 13% and 57% of patients were willing to pay for a pharmacy service, depending on the type of the provided service (10). A recent study showed that 32.1% of patients were willing to pay one of the pharmacy services aimed at asthma, dyslipidemias, or diabetes (11). However, other studies presented much higher percentage of patients who were willing to pay for certain pharmacy services. For the pharmacy service that reduces the risk of drug- related problems by 40% to 20%, 60% of responders were willing to pay (12). Another study showed that more than 85% of women were willing to pay at least US$20 for pharmacist-provided consultations on menopause and hormone replacement therapy (13). Similar monetary value (30 AUD) was obtained in the Australian research regarding the diabetes therapy management service in community pharmacies (14). For the medication therapy management services, Medicare patients were willing to pay US$33 (15). For the same service, patients in Canada were willing to pay lower amount (16). On the other hand, for less complex pharmacy services, such as dispensing, patients were willing to pay much smaller amounts of money (5). Less complex services were not included in our study because they are part of usual pharmacist activity in community pharmacy.

In our study, the willingness to pay for pharmacy service closely correlated with higher respondents’ education, respondent’s medical background, pharmacist being the main source of the information on medicines, and respondent’s need for the service. Respondent’s age did not correlate with the willingness to pay for cognitive pharmacist service, which is in line with previous findings (12-14,17,18). Similarly, the gender of the responders had no influence on the willingness to pay, as shown previously (12-14,17). Our findings on responders’ attitudes and the main source of medical information were comparable to the results from the 2001 National American Pharmacy Consumer Survey (18). Convenience was the primary motive for visiting a specific pharmacy, followed by the product price and pharmacist service and participants ranked physicians and pharmacists as the first and second most important source of health-related information.

One of the rare studies that evaluated consumer rationale for not purchasing medication therapy management service showed that the lack of financial resources was the most common reason (11). More than one-third of the respondents in our study expressed financial concerns, followed by the perception that the service was already provided by other health care professional or that these services were not needed. Financial concerns were most common in asthma management service, while the most common reason for dyslipidemia management service were the respondent’s perception that the service was already provided by other health care professionals.

In our study, the change in the respondents’ willingness to pay closely correlated with the percentage of out-of-pocket payment. Findings from the study by Schuh and Droege (19) suggested that less than half of patients were willing to pay 100% out-of-pocket for cognitive pharmacist service. However, in case of the co-payment with the health insurance, the percentages of patients willing to pay were much higher, reaching 70.2% in case of 20% co-payment, or 84.7% if insurance completely covered the service. In our study, 38% of respondents reported willingness to pay for service provided in the community pharmacy, which may be explained by lower average salary in Serbia compared with the more developed countries.

Currently, in most countries, payment for pharmacist services is limited to the dispensing of the medicines or medical devices, with very little or none fee for the pharmacist cognitive service. In literature, there is a growing body of evidence on different models or mechanisms for sustainable financing and reimbursement of pharmacy services (20-22). The pharmacist contribution in improvement of health care, clinical, and humanistic outcomes of the patients has been well documented and primarily reflected in the knowledge, expertise, and accessibility of the pharmacist as health care professional (21-24). In addition to the fact that patients recognize the pharmacist as medication expert, the importance of pharmacist cognitive services in the health promotion and disease prevention is not fully recognized by the patients. This can be explained by the insufficient patient knowledge, lack of awareness for the services provided, lack of separate counselling space in pharmacy, or lack of time (10,16,24,25). On the other hand, the physicians clearly appreciate the pharmacist contribution at primary health care level (10,21,26). Beside the pharmacist’s contribution to patient’s clinical outcomes, physicians place much higher value on pharmacist service in monetary terms (26).

Wang and Hong (27) evaluated the pharmacists’ willingness to accept a medication therapy management service and found that the pharmacist stated compensation level was significantly higher than compulsory compensation level or patients’ willingness to pay. Surprisingly, even in case of legislative opportunity for the compensation of the service (21), only 54% of pharmacist reported billing for provided service (28). The most common reason for not billing was “indigent population”, closely followed by “salaried position”, “company management does not support charging patients” and “lack of billing standardization”.

Although our respondents were willing to pay relatively low monetary value for pharmacy service in Serbia, results should be carefully considered in the context of country economic development and the purchasing power of the general population. Also, our results may not be easily transferred and extrapolated on other pharmacy services or services provided in hospital setting. As a rule, much higher percentage of patients are willing to pay for pharmacy services provided in hospitals with significantly higher level of compensation compared to services provided in community pharmacy. The same applies to complex, comprehensive pharmacist services. Even if the setting of the service was changed, eg, from hospital to treatment at home, patients would be willing to pay higher value for service (29).

The limitations of our study are typical. The willingness to pay studies determinate only the respondents’ claim that they would pay for the certain service or product, which does not necessarily imply that that would be the case in reality. This effect is named hypothetical bias. Studies covering this type of issues showed that application of the contingent valuation questionnaire resolves this issue (30-32). Also, since the study was designed as cross-sectional, it was impossible to determine the causality.

In conclusion, our study showed the willingness of respondents to pay for cognitive pharmacist service. Our results may provide additional supporting evidence to third party payers for the remuneration of those types of services. In order to obtain a wider perspective, the future research should focus on models of funding and practical implementation of the cognitive pharmacy service.
